# Cases of Hydrocephalus or Water in the Head

**Published:** 1840-01

**Authors:** 


### ART. XI.

*Cases of Hydrocephalus, or Water in the Head; with Observations, and a Detail of a successful Plan of Cure.* By J. F. BARNARD, M.R.C.S. —*Bath,* 1839. 8vo, pp. 30.

MR. BARNARD has exposed himself, in the most marked way, to the severity of criticism, by the manner in which he has chosen to present this pamphlet to the world. To say nothing of the "popular" style of the title-page, what justification can he offer for totally suppressing all reference or allusion to authors who proposed and practised his "new and successful plan of cure" before the earliest of his cases was given to the world? Mr. B. cannot even plead ignorance of this fact; as the authors alluded to were severally pointed out to him in the Lancet of last year, in an annotation on one of his own communications. It seems hardly possible, after reading the whole pamphlet, to come to any other conclusion than that the author wished to make the public believe that his practice was both new and original, when he must know that it is neither the one nor the other.

In the notice in the Lancet (p. 376) both Riverius and Rosen are referred to as having recommended or employed compression of the head in chronic hydrocephalus; but it is chiefly to the late Sir Gilbert Blane (also referred to in the Lancet) that we are indebted for calling attention to the practice in recent times, and for enforcing it both by precept and example. In the London Medical Journal for October, 1821, Sir Gilbert (who appears to have been unaware of the measure having been previously recommended by others) published a short paper "on the effect of mechanical compression of the head, as a preventive and cure in certain cases of hydrocephalus," which, like all the productions of that admirable writer, is marked by a happy admixture of ingenious but cautious theory and sound practical sense. As the notice taken of Mr. Barnard's pamphlet by the periodical press will probably call renewed attention to the subject, we shall here give some extracts from Sir Gilbert's memoir, previously to noticing the contents of the work before us.

Sir Gilbert, as already said, thought his proposal original, and justly regarded it as the more interesting, from its being one of the few practical truths that have been discovered on suggestions derived by physiological theory.

"In reflecting on the circumstances which characterize the history and description of hydrocephalus, some of the chief of which are, that it is very seldom met with but in very early life, and most commonly in infancy before the bregma is closed—that there is in most cases a preternatural size of the head, and that it is usually attended with a rachitic state of the bones and a general scrofulous flaccidity of the soft parts, and runs in particular families; it occurred to me that the distention of the head and bregma is owing to a want of firmness and due resistance in the bony *compages* of the skull, which consequently yields to that effort of pressure with which the brain in its growth acts on its parietes. In reasoning further on the subject, it appeared to me conformable to some of the most approved principles of physiology, that, as there is a certain degree of tension and pressure necessary to the sound condition and action of parts, the withdrawing of this, by inviting afflux and congestion, produces serous effusion; and that for the like reason, there may be a deficiency of that interstitial absorption upon which the healthy state of this and all other soft parts of the living frame depends.

"It was reflections of this kind, whether well or ill founded, which some time ago suggested to me that mechanical compression of the head might be of use in the cure of hydrocephalus, and which induced me to make trial of it in a case which occurred to me last year (1820). A child, aged thirteen months, had a head of a preternatural size almost from birth, and the bregma was preternaturally large. The conformation of the child was otherwise defective; for there was a visible curvature of the spine, indicating a rachitic diathesis. He had for several months been subject to drowsiness; and latterly it was evident, from his screaming, and from raising his hand to his head, that there were occasional paroxysms of headache. There had also been for some time past a dilatation of the pupils. The functions of the bowels were not so much disordered as is generally the case in this disease, which was still in an unformed state.

"I directed the head to be swathed with a roller, as tight as could be done without producing pain or uneasiness. The only other remedies were three leeches, applied once only to the temples and forehead, and a purge every two days of rhubarb and sulphate of potash: mercury was not used in any form. An immediate amendment took place, and continued, so that all complaint was removed in less than three months, except the curvature of the spine; and he has continued well till now, that is, for eighteen months.

"The only other case which has since occurred in my practice has been one which did not offer so fair a field for the trial. The subject was a child of three years of age, with a head preternaturally large, and the bregma not yet closed. No symptoms of hydrocephalus had appeared, but only a state of general delicacy. The swathing has apparently been of benefit, and may probably prevent a disorder naturally to be expected from such a conformation."

This paper was reprinted in 1822, in the author's "Select Dissertations," with the addition of two short but very important communications from friends, confirming the success of the practice. The first from Mr. Costerton, of Yarmouth, and was clearly a case of constitutional hydrocephalus. The child was six months old, and the head had been enlarged three months. Dr. Girdlestone proposed the treatment to Mr. C. on reading Sir G. Blane's paper:

"Dr. Girdlestone wished me to try first, without the aid of medicine, the simple effect of pressure on the brain; and as I could not succeed in confining a bandage round the parts, I put a double strap of adhesive plaster round the head, which completely answered the purpose. The straps remained firm as they were originally fixed. The child gradually improved under the pressure; and the head, which was originally bald, began to be covered with hair, and to acquire more uniformity; and as the muscular strength of the abdomen increased, the ruptures disappeared. The teeth are cutting, the child is growing stronger, and may be said to be nearly well."

The other communication is from Dr. Thackray, of Cambridge, which we shall give in full:

"I am indebted to you for the idea of bandaging the heads of children, where that organ is larger than usual, and when the sutures do not seem ready to close in the usual time. In two instances I have seen great benefit from it. In one, where there seemed every proof of water being accumulated in the ventricles, the bandage seems to have effected a complete cure. The child (thirteen months old) had never been able to sit up, and was easy only in the horizontal posture, was seized with fever, screamings, and most horrid squintings, and very spinage-like stools, Leeches often repeated, and calomel in large doses, reduced the fever, but the squinting and inability to sit up continued. I now tried a bandage of adhesive plaster around the head, and almost instantly the *strabismus* left the child, and she has gradually progressed to a firmness of muscles, can now sit in the nurse's arms, and can bear to be danced about. She still wears the bandage, which has now been on above six weeks. I conceive the child owes its life to this practice."

It is curious that both his correspondents were led to adopt the use of adhesive straps in place of the cloth bandages proposed by Sir Gilbert Blane: this he states to be "a judicious improvement on his method."

In October, 1823, exactly two years after Sir G. Blane's first publication, the author of the pamphlet before us communicated another very interesting case in which the same treatment proved successful. This we shall give at length, as now republished:

"A child, about a year and a half old, was born to all appearance perfectly healthy, and continued so until six months old, when the head was first observed to increase in size. I did not see it until the disease was so far advanced that I almost despaired of its terminating favorably. The head was exceedingly large, weighing, I should think, nearly as much as two thirds of the rest of the body, and measuring in circumference twenty-two inches and a half. The child lay in a state of stupor, and was unable in the least degree to move its head. There was slight strabismus and a rolling of the eyeballs, and almost constant startings of the muscles of the whole body, but more particularly of the face. The countenance had a cadaverous appearance, and the skin was of a yellowish colour. The eyes were sunk in their sockets, and inclosed in a dark ring. The flesh was flabby and seemingly hanging on the bones. The evacuations from the bowels were particularly unhealthy, sometimes green, sometimes blackish, but never of a healthy colour, nor indeed had they been healthy since half a year after its birth. The tongue was constantly covered with a thick white coat; when its head was moved it screamed, and seemed sensible of pain. I expected the child would survive but a few days. I therefore communicated my opinion to the parents, and told them the only chance I saw of saving their child was a plan which I shall describe, and which they readily assented to. I should say that it had been taking purgatives and mercurials without benefit before I saw it. I directed the head to be shaved perfectly clean, I then applied strips of adhesive plaster, about three quarters of an inch wide, completely round the head from before backwards, and so that the ends overlapped each other two inches behind, and covering the space from the eyebrows to where the hair commences, and as low down as the ears would permit; then, with cross strips, from one side to the other, over the crown of the head; and, lastly, one long strip, reaching from the forehead within half an inch of the root of the nose over the crown of the head, likewise to the nape of the neck. This gave effectual support to the parietes of the cranium. I ordered the whole head to be kept constantly covered with linen dipped in cold water, and that the child should take no other medicine than a little castor oil, should the bowels require it. Having thus decided on my practice, I patiently waited the result. Its good effects were evident; in less than a week the little patient could move its head much better, the squinting had disappeared, the secretions from the bowels were more healthy, and the startings of the muscles were less frequent. He had not screamed on rolling or moving the head since the bandage was applied. In a fortnight, the size of the head was reduced in circumference three quarters of an inch; the child was more lively and began to take notice of the persons around it; the secretions from the bowels were perfectly healthy, and evacuated regularly; the tongue nearly clean, and the skin of a natural colour; the countenance more composed and animated. Two months after the bandage was first applied, the child appeared in every respect healthy, but the head was still larger than it ought to be—measuring twenty inches and rather more in circumference; the flesh was firm, and the skin of a healthy mottled hue. The bandage was worn about two months longer, having been renewed about once a fortnight. The bones were then united, and the head firm, and the child well, only requiring time to bring its muscles into action which had been so long quiescent."

The practice detailed in this case is that adopted in all the other cases, with the omission of the cold water. Five more cases are detailed, characterized by similar symptoms and having the same favorable issue: and it would appear that Mr. Barnard's experience could furnish many more. " It would be useless (he says) to multiply cases which are so similar in their symptoms, progress, and cure, but here are sufficient to show the efficacy of a practice which I have never known to fail." Now, we think a tabular view of the cases, showing their nature, the name and age of the subjects, duration of the treatment, &c. &c., would have been by no means useless, and infinitely more satisfactory than the meagre and sweeping statement we have quoted. Mr. B. also refers to ten cases successfully treated by Dr. Engelmann, and one noticed in our last Number by Dr. Löwenhardt.

We are thankful to Mr. Barnard for lending his aid to call the attention of the profession to this very important mode of treatment, and we only regret that his merit in so doing is so greatly allayed by the unhappy manner in which he has put forth his experience.

